# A five year trend analysis of malaria prevalence in Guba district, Benishangul-Gumuz regional state, western Ethiopia: a retrospective study

**DOI:** 10.1186/s40794-020-00112-4

**Published:** 2020-09-09

**Authors:** Shemsia Alkadir, Tegenu Gelana, Araya Gebresilassie

**Affiliations:** Department of Zoological Sciences, College of Natural and Computational Sciences, Addis Ababa University, Addis Ababa, Ethiopia

**Keywords:** Ethiopia, Guba, Malaria, Prevalence, Retrospective

## Abstract

**Background:**

In Ethiopia, malaria is a serious public health concern and has great impact on socio-economy. The trend analysis of malaria data from health facilities is useful for understanding its transmission dynamics and implementing evidence-based malaria control strategies. The aim of this study was to determine the trends of malaria infection in Guba district, western Ethiopia.

**Methods:**

A retrospective study was undertaken at Mankush Health Centre, western Ethiopia. All malaria cases reported from 2014 to 2018 were carefully reviewed from the laboratory record books to determine the trends of malaria morbidity. Data were analyzed using SPSS version 20.0.

**Results:**

In total, 16,964 malaria suspects were diagnosed using microscopy over the last 5 years, of which 8658 (51.04%) were confirmed positive cases. *Plasmodium falciparum*, *P. vivax*, and mixed infection (both species) accounted for 75.2, 24.5 and 0.28% of the cases, respectively. Males patients were more affected (*n* = 5028, 58.1%) than female ones (*n* = 3630, 41.9%). Of the total confirmed cases, 60.4% were age group of subjects (≥ 15 years) followed by 22.6% of 5–14 years and 15.9% of under 5 years. High malaria prevalence was observed in spring (September to November) season, while the least was observed in autumn (March to May) with the prevalence of 45.6 and 11.5%, respectively.

**Conclusions:**

The study demonstrated that malaria is a public health concern, in which *P. falciparum* is the predominant species followed by *P. vivax*. Therefore, the district health bureau and other concerned stakeholders should strength evidence-based malaria control and prevention interventions to interrupt disease transmission and eventual reduction malaria of malaria cases in Guba district.

## Background

Malaria morbidity and mortality in Ethiopia have been profoundly reduced over the last two decades following improved coverage of key malaria interventions throughout the country [1, 2]. Despite these gains, malaria still remains the leading cause of outpatient visits, health facility admissions, and inpatient deaths in the previous years (FMOH, 2012). In 2016, there were an estimated 2,927,266 new malaria cases and 4782 deaths [3]. In addition, 30% of the overall disability adjusted life years (DALYs) are lost, making it a significant impediment to social and economic development [4]. *Plasmodium falciparum* and *P. vivax* are the most predominant and widely distributed parasites in Ethiopia, constituting 60 and 40% of malaria cases, respectively [5].

Around 68% Ethiopian landmass is considered endemic for malaria, putting 60% of the total population more at risk of contracting the disease [6]. The levels of malaria risk and transmission intensity show marked seasonal, inter-annual and spatial variability; with the exception of the southwestern low land area where transmission is year-around [7]. In most regions of Ethiopia, the transmission of malaria peaks from September to December following heavy summer rainy season and lower transmission lasts from April to May following short rainy season [1, 6]. Also, prevalence and incidence of malaria vary depending on variations in socio-demographic risk factors, including age and sex [8]. The unstable transmission patterns along with environmental modifications often make the country prone to cyclic epidemics occurring every 5 to 8 years [1, 6]. For this reason, monitoring malaria burden and trend in endemic areas is critical to monitor to measure the impact of interventions. However, such useful data remain scarce in several endemic areas of Ethiopia, particularly in parts of Benishangul-Gumuz Regional State.

Risk of malaria is high within the Benishangul-Gumuz Regional State, in which 98% of the landmass is prone to malaria transmission [9]. For instance, three years malaria surveillance data from different districts of the region revealed that total prevalence of malaria and malaria specific deaths were 57.5% and 79 deaths, respectively [10]. Another study in Yasso district within the region also revealed that 75% of the study subjects were positive for malarial parasites [11]. Strikingly, a total of 54,197 malaria cases and 7 deaths were recorded in Menge district, Benishangul Gumuz from July 2013–June 2017 [12]. In spite of the general public health significance and the widespread incidence of malaria in several districts of the region, the general trend of malaria prevalence has not been thoroughly studied in Guba district in particular. Analyzing the morbidity pattern of malaria in endemic areas of malaria would help to understand the dynamics of disease transmission and to evaluate the effectiveness of malaria interventions options to curb the disease burden in a locality. Therefore, this study aimed to assess the trends of malaria and transmission pattern over sex, age and seasons for the past five years at Mankush Health Center, western Ethiopia.

## Methods

### Study area

Retrospective study was conducted in Guba district of Benishangul-Gumuz Regional State, western Ethiopia. Mankush Health Center is found in Mankush town, capital of Guba district, located 811 kms west of Addis Ababa. The district lies at between 34°30′ to 37°15′E longitude and 10°54.8′ to 12°22′N latitude. The area had a mean temperature of 26 °C and an annual rainfall of 1323 mill liters over the last 20 years (Ethiopian Meteorology Agency, unpublished data). The district has an estimated total population of 10,851, of whom 5305 are men and 5546 are women [13]. In Guba district, there has been high influx of migrant labors from various areas of Ethiopia due to intensive agricultural activities currently undergoing. In addition, large numbers of young people come to the district in search of employment as construction labor forces in the Great Ethiopian Renaissance Dam (GERD) construction project, where much of the reservoir area of the dam is located.

Both public and private health facilities provide healthcare service to the community in the district, which include 2 health centers, 15 health posts, and 3 primary level private clinics. Malaria diagnosis service has been provided in all public health facilities; however, during the study period parasitological diagnosis service was only done at Mankush Health Center. Patients clinically diagnosed at these health centers, and malaria suspected individuals, visiting this particular health center constituted the catchment population for Mankush Health Center. Accordingly, catchment population of around 8500 is served by this health center annually Seemingly, this was the only health facility that had five years of complete records of malaria case patients. Thus, the present study primarily focused on analyzing the five year malaria trends at Mankush Health Center in Guba district.

### Study design and population

A health facility-based retrospective study was conducted to determine the trends of malaria burden during the past 5 years at Mankush health center. The target populations for the study were all malaria suspected individuals who had visited the health center from January 2014 to December 2018. However, many febrile cases that that possibly received treatment either from health extension workers or self-medication were not included.

### Data collection

A five year (2014–2018) retrospective data on the trend of malaria prevalence were carefully reviewed from the institutional malaria registration book. The parameters such as date of examination, total clinically treated and confirmed cases in months and years, types of malaria species, and socio-demographic data such as age and sex were collected. Data was collected by experienced medical laboratory technicians. Any data such as the socio demographic, and malaria diagnosis results which were not properly documented were excluded. Throughout the reviewed period, microscopy was used as the gold standard for the detection and species identification of *Plasmodium* parasite by examination of peripheral smears of stained blood films, as per the WHO protocol. The health center strictly follows the standard operating procedures for capillary and venous blood sample collection, smear preparation, staining and blood film examination for malaria parasite detection in all phases of the quality control. The blood slides were read and then classified qualitatively as either negative, *P. falciparum* positive, *P. vivax* positive, or mixed infection. Patients with positive peripheral blood smears were offered anti-malarial treatment as per national guidelines [14].

### Data quality control

The completeness of the malaria registration books in the health center was first assessed to ensure the quality of data. Then, data collection format sheet was prepared and used for data recording. Prior to data extraction, data collectors were adequately trained about the data extraction. The overall process of data extraction was followed up by the investigators, where a sample of completed data collection form was randomly selected and checked daily for accuracy, completeness, and consistency. We have also checked the number of confirmed cases with the number of suspected cases throughout the reviewed data.

### Statistical analysis

Data were entered to Microsoft office excels worksheet 2007 and analyzed using SPSS version 20. Descriptive statistics was employed to calculate frequencies and percentages of overall malaria prevalence, trends of malaria transmission in terms of seasons, years, sex, age and species of malaria parasite. Chi square test was used to compare the, association of malaria burden by sex and age groups. Altogether, *P < 0.05* was considered as statistically significant. The analyzed data was presented using tables and figures.

## Results

### Annual trends of malaria burden

**Fig. 1:**
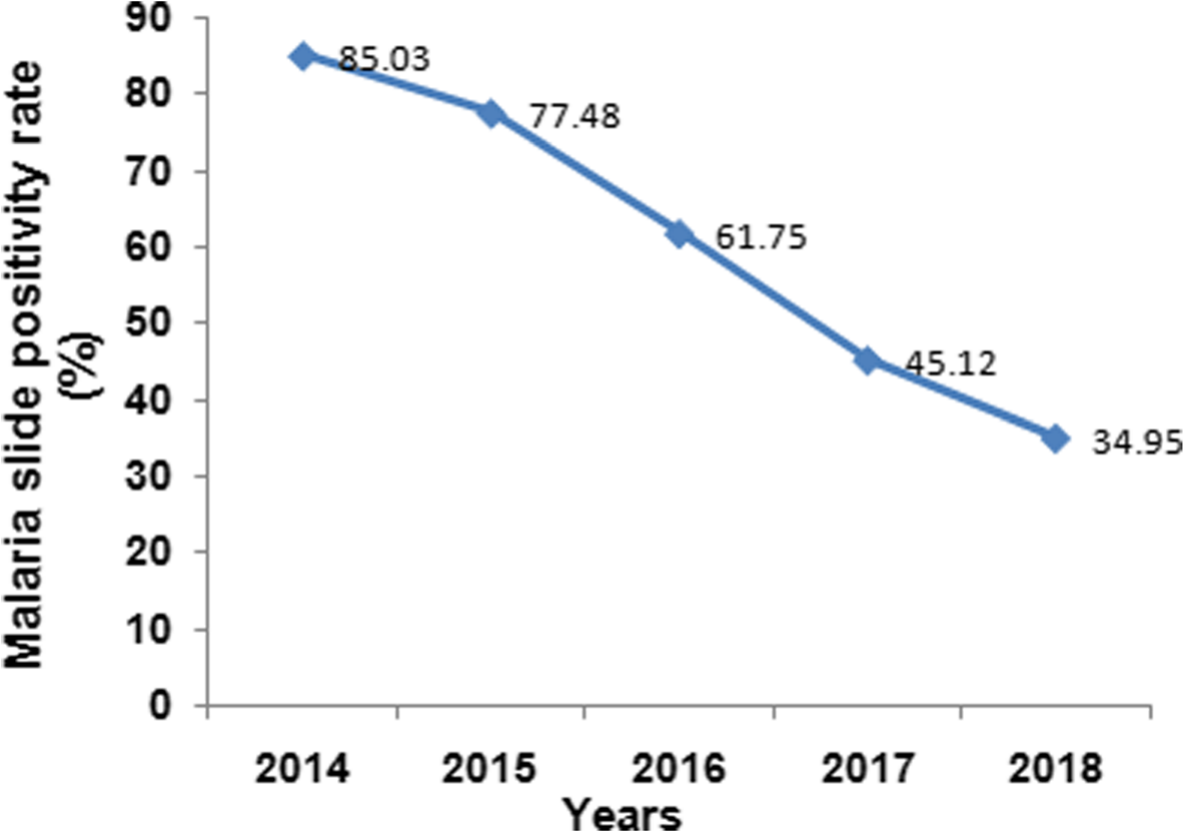
Trend of blood film positive rate of malaria in Mankush Health Center, western Ethiopia: (2014–2018)

Within the five years (2014–2018), a total of 16,964 malaria-suspected patients were diagnosed at Mankush Health Center (Table 1). Out of these, 8658 (51.04%) were microscopically confirmed malaria cases. Annually on average, 1732 malaria confirmed cases were recorded. The number of malaria suspected cases progressively increased from 2014 to 2016, and then it sharply grown during 2016 to 2018 (Table 1). The highest prevalence of malaria cases was observed in the year 2014 in which 1352 patients were positive out of 1590 visitors corresponding to 85.03%. A comparative but lower prevalence was also observed during the next year of 2015 with rate of 77.48% (1424 cases out of 1838). Overall slide positivity rate declined from 85.03% in 2014 to 35%% in 2018 (Fig. 1).

**Table 1:** Annual trends in total malaria cases in Mankush Health Center, western Ethiopia: (2014–2018)

Year	Blood films examined	Lab confirmed malaria cases
2014	1590	1352
2015	1838	1424
2016	2557	1579
2017	4581	2067
2018	6398	2236
Total	16,964	8658

### Prevalence of malaria cases in relation to sex and age

Table 2 shows the number of confirmed malaria cases by sex over the past 5 years. A statistically significant variation (*χ2 = 284.15, d.f. = 1, P < 0.05*) in malaria prevalence was observed between sex. Out of 8658 confirmed malaria cases, 5028 (58.1%) and 3630 (41.9%) were reported in males and females, respectively, with a male to female ratio of 1.39. For each year, the numbers of malaria-positive males were higher than that of females.

**Table 2:** Distribution of malaria cases by sex at Mankusha Health Center, northwest Ethiopia: (2014–2018)

Sex	Total cases examined	Slide positive No. (%)	*P. falciparum* No. (%)	*P. vivax* No. (%)	Mixed No. (%)
Male	9778	5028 (58.1)	3767 (74.92)	1246 (24.78)	15 (0.29)
Female	7186	3630 (41.9)	2746 (75.64)	875 (24.07)	9(0.25)
Total	16,964	8658	6513 (75.22)	2121 (24.49)	24 (0.28)

### *Plasmodium* parasite distribution with age groups

**Fig. 2:**
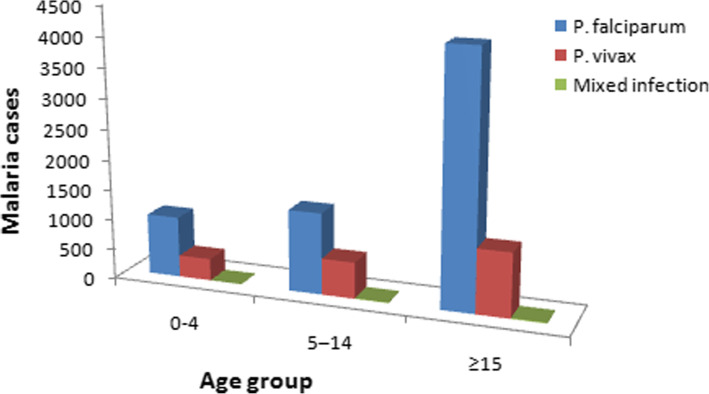
Distribution of *Plasmodium* species by age groups in Mankusha Health Center, western Ethiopia: (2014–2018)

The distribution of parasite species in relation to age is shown in Fig. 2. There was a statistically significant association between malaria burden and age groups (*χ*^*2*^ *= 133.0, d.f. = 2, P < 0.05*). Age groups ≥15 years were more affected, with a prevalence rate of 5228 (60.4%), followed by 5–14 years old, and under five children with prevalence rates of 1955 (22.6%) and 1375 (15.9%), respectively. In relation to *Plasmodium* spp., *P. falciparum* was the predominant parasite in all age groups, and it was higher in age group ≥15 years, and 5–14 age groups with a prevalence rate of 4155 (79.5%) and 1346 (58.4%), respectively. The age groups ≥15 was more affected by *P. vivax* 1058 (20.24), followed by 5–14 year olds, 600 (30.7%) and below 5 year 363 (26.4%).

### *Plasmodium* species distribution

**Fig. 3:**
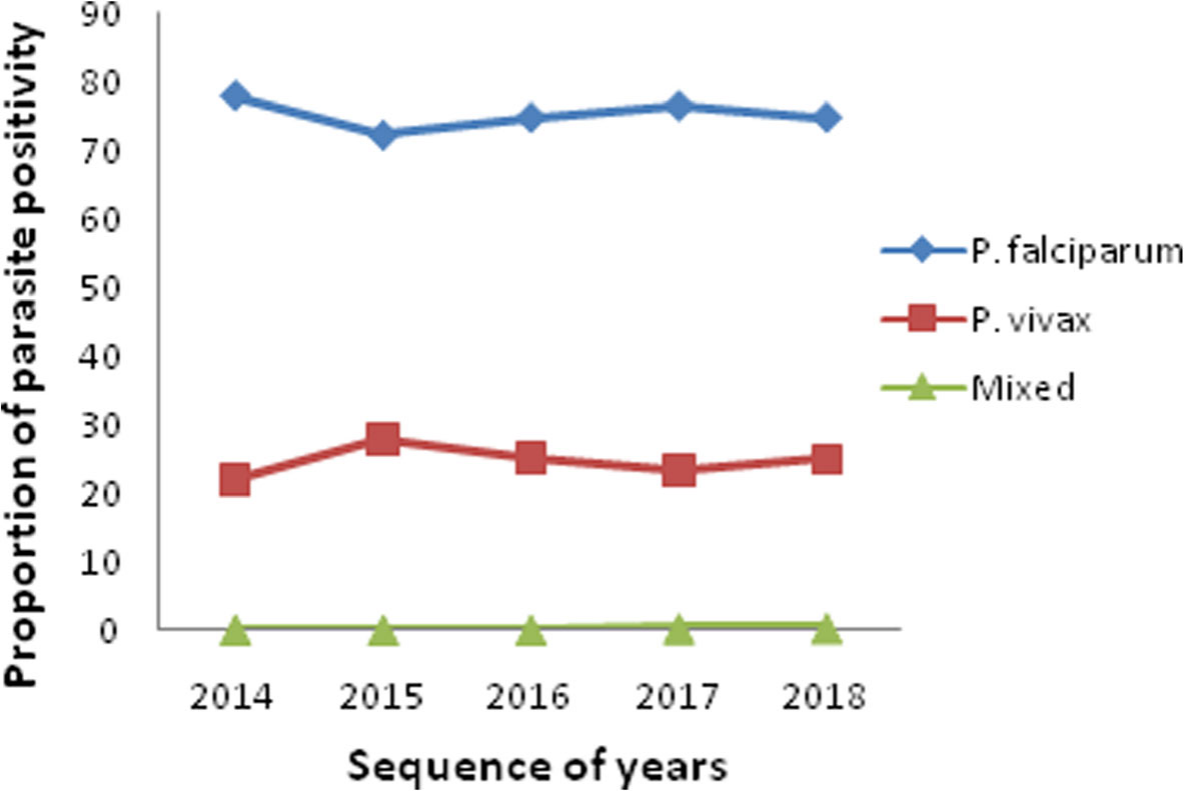
Species trends of malaria parasites in Mankusha Health Center, western Ethiopia: (2014–2018)

*Plasmodium falciparum* and *P. vivax* were the only species in study area, where *P. falciparum* accounted for 6513 (75.23%) of the overall prevalence, followed by *P. vivax* constituting 2121 (24.50%). Mixed infection (*P. falciparum* + *P. vivax*) accounted only for 24 (0.28%) of the total cases. In the five year trend, *P. falciparum* was threefold more dominant than *P. vivax* (75.23% vs. 24.50%). The prevalence of *P. falciparum* slightly declined from 77.96% in 2014 to 74.74% at the end of 2018, (Fig. 3). However, there was a slight increment in the proportion of *P. vivax* from 21.82% in 2014 to 24.82% in 2018, with peak prevalence rate of 27.74% in 2015.

### Seasonal distribution of malaria

**Fig. 4:**
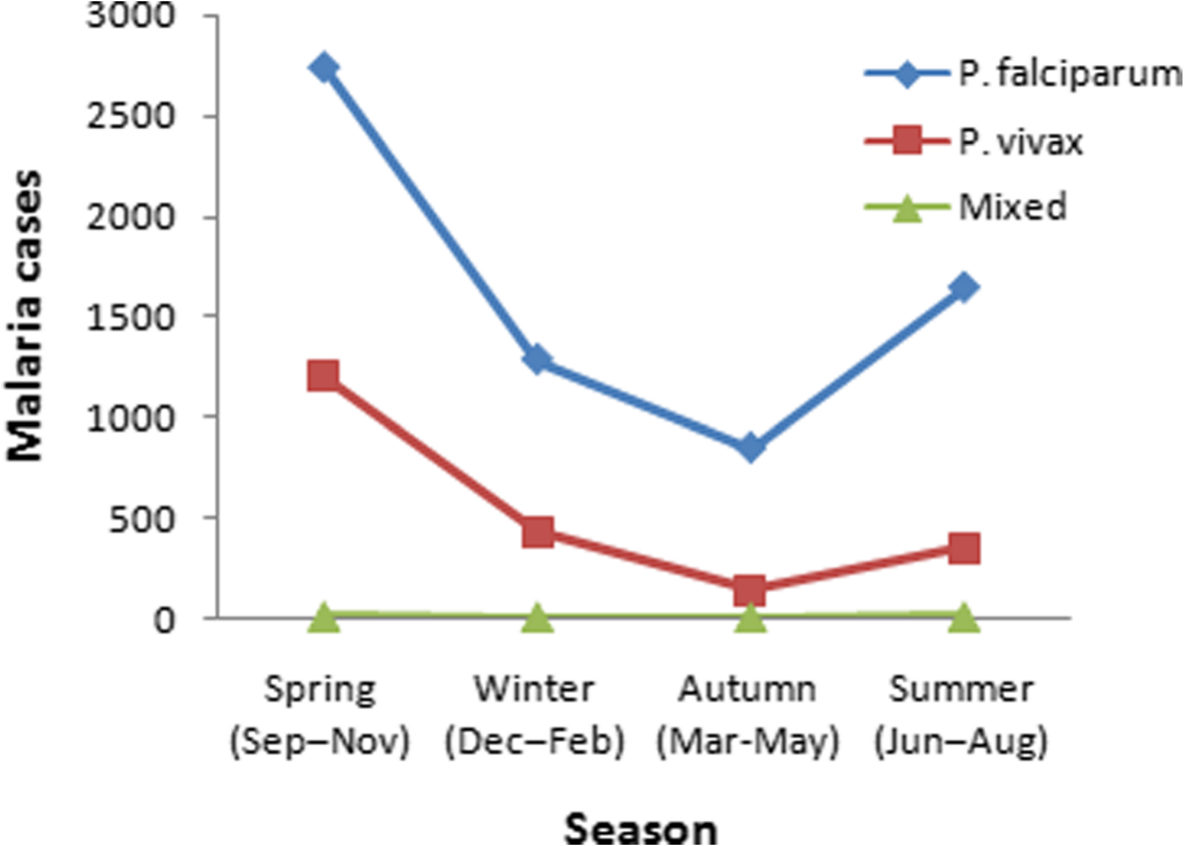
The distribution of *Plasmodium* species in different seasons in Mankush heath center, western Ethiopia from 2014 to 2018

The seasonal distribution of malaria cases is presented in Fig. 4. Though malaria occurred in all seasons, the prevalence had fluctuating trend across the four seasons over the last 5 years. The highest and the lowest cases of malaria were observed during spring (September, October and November) (45.6%) and autumn (March–May) (11.5%), respectively. Higher number of cases of *P. falciparum* was observed in spring and summer, while more cases of *P. vivax* were observed in spring, followed by winter (Fig. 4). However, the minimum number of *P. falciparum* and *P. vivax* cases were observed during autumn (March–May).

## Discussion

The overall prevalence of malaria in this study was 51.04% where a total of 8658 confirmed malaria cases were detected in five years retrospective study, important indicator for existence of malaria burden in Guba district. Such higher malaria burden demands concerted efforts on deployment of effective malaria prevention and control strategies. This higher malaria positivity rate detected in this particular study is comparable with the three years malaria surveillance report of the Benishangul Gumuz Regional State that reported 57.5% [10]. However, markedly higher overall malaria positivity rates was detected compared to other previous retrospective studies from different parts of Ethiopia, reported a total prevalence rates ranging from 21.8 to 39.6% [15–17]. The observed variations might be attributed to differences in microclimate, altitude, expansion of development projects like dams or irrigation, community awareness about malaria transmission and control, and malaria intervention practices [18–20].

In the present retrospective data analysis, malaria prevalence trend appeared to be non-fluctuating, in which the malaria positivity rate continued to show consistent decline in the past five years. This finding is congruent with similar studies from several malaria endemic areas of Ethiopia which reported a decrease in malaria prevalence from 2001 to 2016 [2, 17, 21, 22]. Ethiopia has been implementing key malaria interventions, including use of insecticide treated net (ITN) and indoor residual spraying (IRS), prompt diagnosis and treatment with artemisinin-based combination therapy, intermittent preventive treatment in pregnancy and environmental management in integrated manner throughout the country since 2005 [1]. Such rigorous activities have also been practiced in Guba district and thus, these interventions possibly resulted in a reduction of malaria cases.

Our data also showed that *P. falciparum* constituted the most predominant malaria infections (75.2%) while markedly lower *P. vivax* was also present (24.5%). In addition, 0.28% of mixed malarial infection of both *P. falciparum* and *P. vivax* was detected. This finding is in agreement with malaria species distribution in several parts of Ethiopia, which reported the predominance of *P. falciparum* over *P. vivax* [6, 12, 15, 23]. However, these findings are less than similar reports from 10 years data documented in Metema Hospital, northwest Ethiopia (91%) [24], and sub-Saharan African countries, where 99.7% of estimated malaria cases were due to *P. falciparum* [25], which could be related.

The study also revealed higher positivity rate of malaria among males (58.1%) than females (41.9%). This finding is in agreement with other studies conducted in various areas of Ethiopia, which reported higher malaria prevalence in males than females [15, 18, 22, 26]. Such higher malaria prevalence in males is conceived to be attributed to the fact that males are usually engaged in outdoor activities that put them at greater risk of contracting the disease [22, 27].

Concerning distribution of malaria prevalence by age groups, majority of reported cases were in the age group of subjects greater 15 years and above, followed by the age group 5–14 years, and under five children. Such results have been reported by other studies [14, 22, 28], where males in this age groups are more susceptibility to malaria infections. In this rural area, males in the reproductive age groups (≥15 years old) are commonly breadwinners of their families, spending most of their time especially evenings outdoors when the peak biting activity of the infective mosquito is observed [29]. Importantly, the observed lower prevalence of malaria in children under 5 years of age might be because of their less likely exposure to infected mosquito bite due to good awareness and practices of their parents/care takers on malaria control and prevention activities.

The prevalence and magnitude of malaria transmission are mainly determined by environmental, climatic and seasonal factors. In this particular study, the highest cases of malaria were observed during spring (September, October and November). The seasonality observed in the current study is in agreement with studies in different parts of Ethiopia [21–23], all of which revealed high malaria transmission periods corresponding to the months of September, October and November. In most parts of Ethiopia, the main malaria transmission season is from September to December, following the rainiest season from June to September [1]. Variability of rainfall and temperature in each season affects the availability of breeding habitats for mosquito vectors, the length of mosquito larvae development, and the rate of growth of the malaria parasites inside the vector [20, 30]. Similarly, there was a second peak in malaria case during summer (June to August). Possibly, this is due to relapsing behavior of some malaria parasite and irregular rain-full in the area.

## Conclusions

In conclusion, the study demonstrated that malaria remains a public health burden in the area with high slide positivity rate. This would be an important indicative that the area needs due attention and further concerted malaria interventions. The deadly *P. falciparum* appeared to be the dominant *Plasmodium* species, and patients in the age groups of 15 years and above, and males were more infected. In addition, malaria transmission in the area peaks from September to December, coinciding with the major harvesting season. Therefore, health planners need to should strength evidence-based malaria control and prevention interventions to interrupt disease transmission and eventual reduction malaria of malaria cases in Guba district and surrounding areas.

## Data Availability

All data underlying the findings are available from corresponding author on reasonable request. All relevant data are within the manuscript.

## Abbreviations

IRS: Indoor residual spraying
ITN: Insecticide treated net
SPSS: Statistical Package for the Social Science
WHO: World Health Organization
